# Complete and durable regression of leptomeningeal involvement during lorlatinib treatment in a patient with lung cancer

**DOI:** 10.1097/CAD.0000000000001637

**Published:** 2024-07-09

**Authors:** Giorgia Guaitoli, Enrica Martinelli, Lucia Trudu, Isacco Desideri, Pietro Mortini, Stefano Greco, Alessio Bruni, Daniela Greto, Guido Pecchioli, Chiara Chiavelli, Massimo Dominici, Federica Bertolini

**Affiliations:** a Division of Oncology, Department of Oncology and Hematology, University Hospital of Modena; b PhD Program Clinical and Experimental Medicine, Department of Biomedical, Metabolic and Neural Sciences, University of Modena and Reggio Emilia, Modena; c Radiation Oncology Unit, Azienda Ospedaliera-Universitaria Careggi; d Department of Biomedical, Experimental and Clinical Sciences ‘Mario Serio’, University of Florence, Florence; e Department of Neurosurgery, San Raffaele University Health Institute Milan, Milan; f Division of Radiotherapy, Department of Oncology and Hematology, University Hospital of Modena, Modena; g Neurosurgery Unit, Azienda Ospedaliero-Universitaria Careggi, Florence; h Laboratory of Cellular Therapy, Division of Oncology, Department of Medical and Surgical Sciences for Children & Adults, University of Modena and Reggio Emilia, Modena, Italy

**Keywords:** leptomeningosis, lung cancer, oligoprogression, stereotactic ablative radiotherapy, tyrosine kinase inhibitors

## Abstract

Metastatic spread to the central nervous system (CNS) is frequent in anaplastic lymphoma kinase (*ALK*)-rearranged non-small cell lung cancer (NSCLC) and has an important impact on patient prognosis and quality of life. Leptomeningeal involvement may occur in up to 10% of cases of ALK-positive NSCLC. Lorlatinib is a third-generation ALK inhibitor that has excellent CNS penetrability and demonstrated its efficacy both in pretreated and treatment-naive patients. Herein, we present the case of a 34-year-old patient diagnosed with stage IV ALK-rearranged NSCLC who received two lines of ALK inhibitors (crizotinib followed by alectinib) and several courses of brain stereotactic ablative radiotherapy until leptomeningeal involvement was detected. Third-line lorlatinib was then administered, and 2 months later encephalic MRI documented complete regression of the leptomeningeal involvement that is still maintained after 36 months while treatment with lorlatinib is still ongoing with good tolerability. To the best of our knowledge, this is the longer intracranial response reported in the literature, underlining the importance of the most appropriate choice of systemic treatments and their integration with loco-regional approaches to improve outcomes.

## Introduction

Most of patients suffering from anaplastic lymphoma kinase (*ALK*)-rearranged non-small cell lung cancer (NSCLC) develop central nervous system (CNS) metastases. Therefore, the intracranial activity of anticancer agents is particularly relevant in this setting, as CNS involvement relates with poor prognosis. Tumor spread to the CNS may also result in leptomeningeal involvement that can occur in up to 10% of ALK-related NSCLC [1]. Lorlatinib is a third-generation ALK tyrosine kinase inhibitor (TKI) that demonstrated its safety and its superiority over crizotinib as a first-line treatment in phase 3 trial [2]. Moreover, a phase 2 study recently reported that lorlatinib achieved a 59% intracranial objective response rate and a median intracranial progression-free survival of 24.6 months among 23 patients who experienced intracranial progression after at least one second-generation ALK inhibitor [3].

Here, we report a case of a young man who experienced an impressive and prolonged response to leptomeningeal carcinomatosis during treatment with lorlatinib after receiving two previous lines of ALK-inhibitors and several courses of stereotactic ablative radiotherapy (SABR) to the brain.

## Case presentation

In December 2017 a 34-year-old Caucasian male was diagnosed with stage IV lung adenocarcinoma with pleural and bone involvement and suspicious brain metastases. No significant comorbidities or previous smoking history were reported. The disease was found to harbor *ALK* rearrangement and first-line treatment with crizotinib (250 mg twice daily) was started. A short 3-month restaging, by contrast enhanced MRI and total body computed tomography (CT) scan, revealed an increase in brain metastases in particular with an increase of a left temporal lesion, while an improvement of other target lesions was observed. The patient was then evaluated by radiation oncologists (RO) who performed SABR, 18 Gy in a single fraction while continuing crizotinib.

In May 2018 new brain metastases appeared and treatment with crizotinib was discontinued. The patient was then addressed to second-line alectinib (600 mg twice-daily). At the time of the first MRI restaging an intracranial partial response was reported, with good tolerance to treatment.

In January 2019, after 6 months of alectinib, a new brain MRI documented stability of the temporal lesion while three other lesions (one right cerebellar and two in the left frontal cortical area) were increased. The patient was then referred again to RO who planned a Gamma-knife treatment. Thus, SABR was performed on these three metastases with a total dose of 24 Gy, without discontinuing alectinib treatment. On April 2019, a brain MRI confirmed the reduction of the treated lesions, but a new small one (diameter 4 mm) was detected in the left temporal lobe, so the patient was submitted to SABR using Gamma-Knife (24 Gy in single fraction).

In September 2019, a spinal MRI was performed due to back pain referred by the patient, and a bone lesion on the second lumbar vertebral body appeared together with paravertebral neoplastic tissue. The patient underwent spinal fractionated stereotactic body radiotherapy (21 Gy in 3 fractions) while continuing alectinib as the disease remained stable in the chest and brain.

**Fig. 1:**
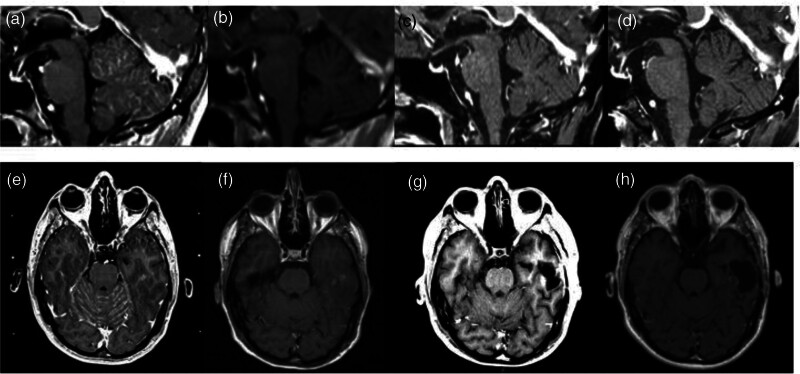
Axial and trasverse brain MRI scans reporting the appearance of leptomeningeal involvement in November 2020 during second-line treatment with alectinib (a,e). After 2 months of treatment with lorlatinib, regression of leptomeningeal involvement was observed (b,f—March 2021) and maintained in subsequent MRI evaluations in February 2022 (c,g) and January 2024 (d,h) after 13 and 36 months of treatment, respectively.

Seven months later (April 2020) the appearance of two new brain lesions was documented, so the patient underwent SABR using Gamma-Knife once again (22 Gy), achieving stable disease until the end of November 2020. At this time brain MRI revealed leptomeningeal involvement (Fig. 1a,e).

As the patient did not present any neurological symptoms and after careful multidisciplinary evaluation, whole brain radiotherapy was postponed and a request for lorlatinib compassionate use was made. In the meantime, alectinib was continued until lorlatinib became available. Third-line treatment with lorlatinib was started at the end of January 2021 at the standard dosage of 100 mg daily. By the end of March 2021, after 2 months of lorlatinib, the regression of leptomeningeal involvement was documented by a CT scan and subsequently confirmed by contrast-enhanced brain MRI (Fig. 1b,f). Of note, a millimetric increase of the lesion in the left temporal lobe which had been previously treated by SABR was observed.

**Fig. 2:**
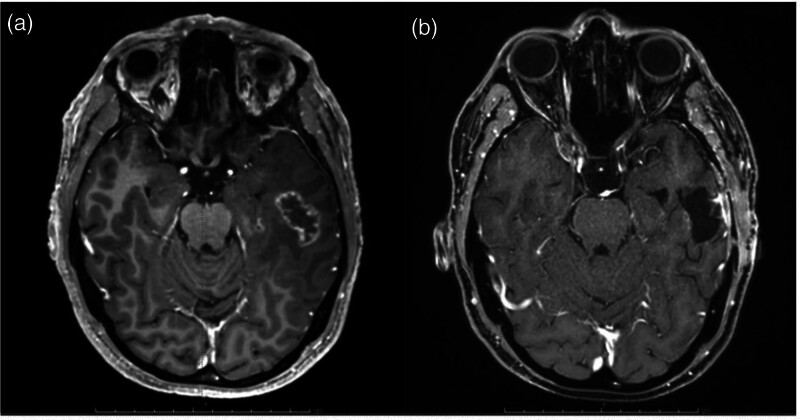
Brain MRI evaluation before (a—November 2021) and after (b—February 2022) the excision of the temporal lesion.

The increase in the size of that lesion was subsequently confirmed by subsequent MRIs and, on November 2021, the patient underwent surgical excision of the lesion without complications (Fig. 2). Interestingly, the histological examination revealed radiation necrosis only, without evidence of neoplastic cells infiltration. Treatment with lorlatinib was discontinued only a few days before and after surgery.

**Fig. 3:**
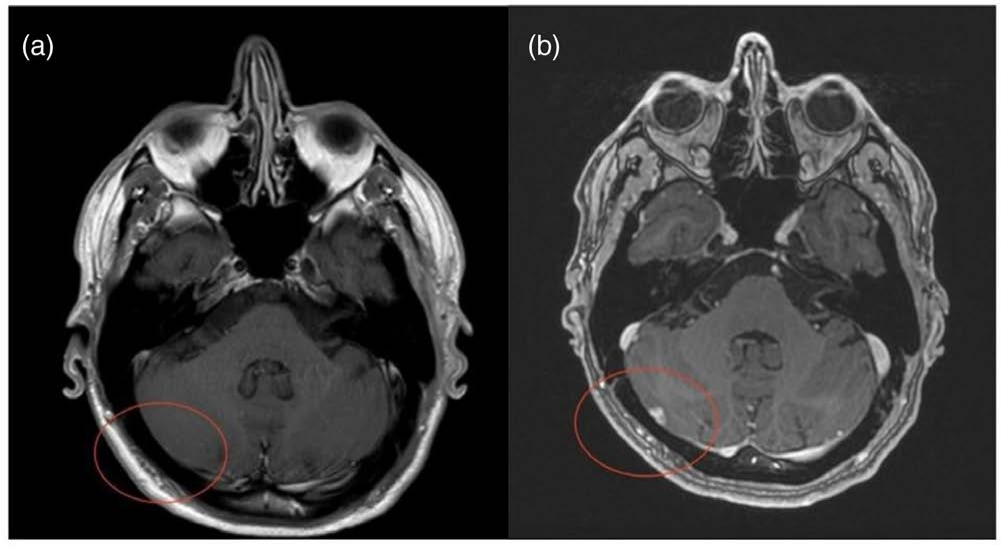
Comparison brain MRI between April 2022 (a) and June 2022 (b) when the cerebellar lesion appeared, after 17 months from the beginning of treatment with lorlatinib.

In March 2022, 14 months from the beginning of treatment with lorlatinib, neither intracranial nor leptomeningeal progression were emerging, while in June 2022 the brain MRI showed a new right cerebellar lesion (diameter 6 mm) (Fig. 3) again managed by Gamma-Knife (22 Gy in single fraction).

Other Gamma-Knife treatments were required in February and May 2023 for the treatment of two lesions in a dimensional increase (one right supramarginal and one left occipital), and a newly appeared right cerebellar lesion, respectively.

**Fig. 4:**
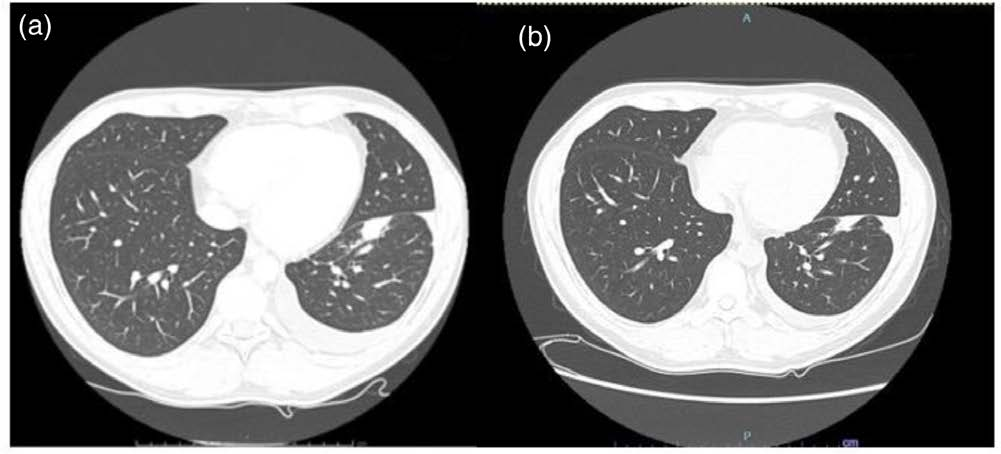
Computed tomography (CT) scans of primary nodule at the beginning of treatment with lorlatinib (a—March 2021) and after 36 months (b—January 2024) showing stable lung disease.

Overall, after 36 months from the beginning of treatment with lorlatinib, the regression of leptomeningeal involvement is still maintained (Fig. 1d,h) with stable lung disease (Fig. 4b). Treatment with lorlatinib is currently ongoing with an impressive encephalic response and, so far, without neurological side effects. The only drug-related side effects reported till now are hypercholesterolemia and hypertriglyceridemia, currently managed by lipid-lowering therapy without reduction of lorlatinib dosage. The patient gave his written informed consent to report his clinical information.

## Discussion

We report an impressive intracranial efficacy of lorlatinib in a patient with *ALK*-rearranged NSCLC who had already received multiple lines of therapy, surgery, and several SABR sessions.

*ALK* rearrangements are a driving oncogene alteration that accounts for about 3–5% of all NSCLC cases [1]. It appears to be more common in younger patients, usually without a smoking history or light smokers.

Lorlatinib is a third-generation ALK inhibitor found to be more effective in crossing the blood-brain barrier, compared with first- and second-generation TKIs [2–5]. It demonstrated its efficacy also in patients who have failed prior ALK TKIs [6]. Zhu *et al*. [7] analyzed 95 heavily pretreated patients with ALK or ROS proto-oncogene 1 (ROS1)-rearranged NSCLC. Among them, 13 (11 ALK+ and two ROS1+) had leptomeningeal carcinomatosis at the beginning of lorlatinib [7]. Intracranial objective response rate, intracranial disease control rate, and median progression-free survival were 45%, 91%, and 9.3 months, respectively. These outcomes are clinically meaningful given the lack of effective therapies for patients with leptomeningeal carcinomatosis.

In this particular case, the patient did not show any neurological symptom, so we could observe only radiological remission. However, cases with important clinical responses have been reported [8,9]. Taketa *et al*. [8] described two cases of patients in which lorlatinib treatment achieved radiological regression, resolution of severe neurological symptoms, and improved performance status. Moreover, a meningeal long-lasting response with lorlatinib in a highly symptomatic patient progressing to second-generation TKI (entrectinib) was described also in one *ROS1*-rearranged NSCLC patient [9].

In addition to the radiological response, we highlight the importance of integrating local ablative treatments (such as SABR) in case of oligoprogression during TKIs treatment, and the safety of this treatment strategy, as already reported in the literature [10].

Regarding the safety profile, in the CROWN trial [2] grade 3 or 4 adverse events were more frequent with lorlatinib than with crizotinib. However, more than half of the grade 3 or 4 adverse events in the lorlatinib arm were represented by increased levels of cholesterol, triglycerides, or both. These side effects can be readily managed with lipid-lowering agents and lorlatinib dose modifications if needed [2].

In conclusion, we reported a very impressive and durable regression of leptomeningeal carcinomatosis in a young patient treated with lorlatinib as a third-line treatment after second-generation TKI progression. To the best of our knowledge, this is the longest regression reported in the international literature. These findings are relevant given the poor outcome of patients with leptomeningeal involvement. In *ALK*-rearranged patients, the development of TKIs with intracranial effectiveness is quite relevant considering the incidence of brain metastases and the young age of most of these patients at the time of diagnosis. Indeed, even in the case of extensive central nervous system involvement, the achievement of drug-induced responses may avoid or postpone the use of large radiotherapy volumes, while stereotactic treatments may maintain their role in the case of limited intracranial oligoprogression. The correct choice of systemic treatments and its integration with multimodal loco-regional approaches are fundamental to improve patients’ outcomes and efforts shall be implemented to create robust evidence in this scenario.

## Acknowledgements

Supported in parts by MIUR Dipartimenti Eccellenti 2017 (GG, MD).

### Conflicts of interest

G.G. reported advisory fees from Roche. Editorial fees from Novartis. Travels and accomodations from AstraZeneca, BMS, MSD and Novartis all outside the current paper. A.B. reported speaker’s fees from Astra Zeneca, Jannssen, Ipsen, and MSD. Travel accomodations from Astra Zeneca, Janssen, all outside the current paper. For the remaining authors, there are no relevant conflicts of interest on the current work.
